# Does cultural background influence the dissemination and severity of the COVID-19 pandemic?

**DOI:** 10.1016/j.heliyon.2022.e08907

**Published:** 2022-02-04

**Authors:** Margarida Duarte, Sérgio Moro, Catarina Ferreira da Silva

**Affiliations:** Instituto Universitário de Lisboa (ISCTE-IUL), ISTAR, Avenida das Forças Armadas, 1649-026 Lisboa, Portugal

**Keywords:** COVID-19, Cultural background, Cultural dimensions, Pandemic dissemination, Coronavirus

## Abstract

The COVID-19 pandemic has spread throughout the globe affecting countries worldwide. However, several differences have been observed in the number of daily new cases, the COVID-19 reproduction rate, and the severity of the disease in different countries. Previous studies have mostly highlighted government restriction policies to mitigate the pandemic effects as reasons for such differences. This study focuses on 101 countries and proposes that each country's cultural background is also accountable for such differences. We considered the six Hofstede's cultural dimensions (power distance, individualism, masculinity, uncertainty avoidance, long term orientation, and indulgence) and statistically analyzed their correlation with several COVID-19 impact metrics in comparison to several restriction policies. Our results support our claim that national culture influences both acceptance and subsequent adoption of restriction policies and the implementation by each government of those policies. We highlight that the attitudes towards and trust in political institutions, policies and governance is influenced by the cultural background, which is reflected in the pandemic numbers. As a main takeaway from this study, we conclude that data-driven models which aim at predicting the pandemic impact evolution at a global scale should also include variables that reflect the cultural background of each nation.

## 1 Introduction

The COVID-19 pandemic has severely affected societies worldwide, causing havoc in healthcare systems as it swiftly swept across the globe (Gaddy, 2020). Such health crisis event happens in a world hosting 7 billion humans, made smaller due to efficient transportation systems permitting people to travel to the Antipodes in 24 h or less (Muley et al., 2020). High-density communities provided toll-free highways for the novel coronavirus to quickly spread airborne or through droplet contact (Manigandan et al., 2020). Therefore, to lower the number of COVID-19 patients in healthcare units, national governments in every single nation had to implement several restrictive measures to cut off the virus propagation (Tabery and Pilnacek, 2021). These so-called Non-Pharmaceutical Interventions (NPIs) include international travel controls, facial coverings, restrictions on internal movements and on gatherings, workplace closure, school closure, and stay-at-home order, among others (Galanis and Hanieh, 2021; Kuhn et al., 2021). While many of those measures, especially when combined, have proven successful in reducing the contagion among populations, the degree to which imposing a certain measure results in lowering the number of new cases is heterogenous among the different countries, partially due to the socio-economic context (Haug et al., 2020). Bendavid et al. (2021) found that the stay-at-home order and business closure had a significantly more positive influence on lowering the number of active cases in Spain and England, when compared to Sweden.

The societal impact of NPIs has several facets. By restricting citizens’ mobility, traveling came to a halt (Piccinelli et al., 2021). By closing all sorts of businesses dependent on personal contact such as restaurants and physical retail stores in non-essential businesses (e.g., clothing), an economic crisis immediately emerged (Sukharev, 2020). Workers who could do their job remotely using online tools, were required to do so (Blanchard, 2021). Nevertheless, not everything can be done remotely. Countries heavily dependent upon tourism such as Greece and Mexico were among the most severely affected (Khalid et al., 2021). Unemployment rose to unforeseen levels, and societies demanded activities reopening (Pronk and Kassler, 2020). Countries with higher levels of poverty were among those where uprisings were higher (Elgar et al., 2020). But not only. Also, those countries with more democratic governments were more susceptible to uprisings (Azmanova, 2020). Although existing literature acknowledges some influence of the economic context to turn a society to more willingly accept NPIs (Rosman et al., 2021), it does not fully explain it. China, a densely populated country where the first COVID-19 cases were reported, was able to successfully control the pandemic, reporting very few daily cases after April 2020, the most of which imported (Jin et al., 2021). Both Japan and South Korea, with democratic governments and densely populated, also managed to be more successful in controlling the pandemic (Chen et al., 2021). In comparison, Western democracies in Europe and North America struggled to lower the number of daily new cases, despite the adoption of several restrictive NPIs (Nande et al., 2021).

In this study, we hypothesize that the cultural context plays a role in the acceptance of NPIs. We argue that a nation is able to deal more (or less) swiftly with the pandemic based upon its individuals’ attitudes towards and trust in political institutions, policies and governance. The impact of the NPIs, considering socioeconomic and cultural factors can be an asset to identify vulnerable cultural minority groups that become a hotbed of infection by COVID-19 in order to address their needs and then also to mitigate the spread of the virus (Waitzberg et al., 2020).

Thus, we argue that the cultural dimensions that characterize nations influence both the dissemination and the severity of the COVID-19 disease in their populations because culture fundamentally shapes how people respond to crises such as the COVID-19 (Lu et al., 2021). According to Erman and Medeiros (2021) during the initial period of a pandemic, policy makers should more explicitly consider the cultural attributes of a society along with other important parameters such as demographic characteristics and healthcare system constraints in order to develop more tailored and effective policy responses.

We adopt the widely studied and accepted six cultural dimensions defined and tuned by Hofstede over the last decades (Hofstede et al., 2010), namely power distance, individualism, masculinity, uncertainty avoidance, long term orientation, and indulgence. We know from existing literature that NPIs partially influence COVID-19 impact metrics (e.g., Davies et al., 2020).

Some studies have already added Hofstede's cultural dimensions along with NPIs in order to study their impact on the pandemic but they have focused on data from the first wave of the pandemic. Geert Hofstede proposed six cultural dimensions, defined in the Hofstede Insights website^1^, that translate a nation's background and context, namely: power distance, individualism, masculinity, uncertainty avoidance, long term orientation, and indulgence. Briefly, power distance refers to the degree of inequality that exists between members of a society, uncertainty avoidance relates to a society's level of stress in the face of uncertain situations, individualism refers to a society's members' need to be integrated into primary groups, masculinity reflects the degree of competitiveness between individuals in a society, long-term orientation distinguishes a society focused on the future or on the present and the past, and, finally, indulgence differentiates societies that like to enjoy their leisure time from societies restricted in this aspect. These have been widely adopted in numerous studies to compare countries' culture (e.g., Al-Haddad and Galib, 2020; Moro, 2020). In Gokmen et al. (2021) national cultural dimensions of 31 European countries were related to the rise in the total number of COVID-19 cases per million. These authors obtained that the power distance has a significant and negative effect, correlating with an increase of the COVID-19 cases per million, while the individualism and indulgence have a positive and significant effect, correlating with a decrease in COVID-19 cases per million. In this sense, Güss and Tuason (2021) shows that countries with high Gross Domestic Product (GDP) per capita, high individualistic values and high intellectual autonomy were associated with high number of COVID-19 deaths, while countries with higher collectivistic values were associated with fewer COVID-19 deaths. Additionally, Luu & Huynh (2020) studied the impact of the cultural factor on respect for social distancing measures and obtained that countries with high uncertainty avoidance predict the lowest proportion of people gathering in public.

Therefore, by assessing the correlation of the cultural dimensions and COVID-19 impact metrics such as the number of daily new cases and number of new fatalities, and by comparing the results with similar ones for the different NPIs, we are able to understand if each cultural dimension plays a likewise important role when compared to each NPI.

## 2 Approach

We grounded our experiments on three datasets. We adopted the “Our World in Data COVID-19” (OWDC) dataset^2^ for retrieving the variables listed in Table 1, related to the pandemic impact and demographic indicators. As for the NPIs data, we used the Oxford COVID-19 Government Response Tracker (OxCGRT).^3^ Both have been used extensively by the academic community to analyze different problems related to the COVID-19 pandemic (Hasell et al., 2020; Hale et al., 2021). Finally, to obtain the cultural dimensions for each country, we collect the six Hofstede's dimensions (HOFSTEDE as referred in Table 1 data source).

**Table 1:** Variables used.

Variable	Description	Category	Source
new_cases_per_million	Nr. of daily new cases per million people	COVID-19 metric	OWDC
new_deaths_per_million	Nr. of daily new deaths per million people		
reproduction_rate	The COVID-19 reproduction rate (Rt)		
icu_patients_per_million	Intensive-care Unit patients per million		
hosp_patients_per_million	Hospitalized patients per million		
new_tests_per_thousand	New COVID-19 daily tests per thousand		
population	Nr. of people living in the country	Demographic	
population_density	Population density		
median_age	Population median age		
aged_65_older	Nr. people with an age equal or above 65 years		
gdp_per_capita	Gross Domestic Product per capita		
extreme_poverty	Nr. people in extreme poverty situation		
cardio_vasc_death_rate	Death rate for deaths due to cardio-vascular disease		
diabetes_prevalence	Prevalence of diabetes disease		
hospital_beds_per_thousand	Nr. hospital beds per thousand people		
life_expectancy	Life expectancy (in years)		
human_development_index	Human development index		
power_distance	Power distance cultural dimension	Cultural context	HOFSTEDE
individualism	Individualism cultural dimension		
masculinity	Masculinity cultural dimension		
uncertainty_avoidance	Uncertainty avoidance cultural dimension		
long_term_orientation	Long-term orientation cultural dimension		
indulgence	Indulgence cultural dimension		
C1: School closing	Closings of schools and universities	NPI	OxCGRT
C2: Workplace closing	Closings of workplaces		
C3: Cancel public events	Cancelling public events		
C4: Restrictions on gatherings	Limits on gatherings		
C5: Close public transport	Closing of public transport		
C6: Stay at home requirements	Orders to "shelter-in-place" and otherwise confine to the home		
C7: Restrictions on internal movements	Restrictions on internal movement between cities/regions		
C8: International travel controls	Restrictions on international travel		
E1: Income support	Government is providing direct cash payments to people who lose their jobs or cannot work.		
E2: Debt/contract relief	Government is freezing financial obligations for households		
H1: Public information campaigns	Presence of public info campaigns		
H2: Testing policy	Policy on who has access to testing		
H3: Contact tracing	Policy on contact tracing after a positive diagnosis		
H6: Facial coverings	Use of facial coverings outside home		
H7: Vaccination policy	Policy on vaccine delivery for different groups		
H8: Protection of elderly people	Policies for protecting elderly people		

The variables adopted from the dataset resulting from merging the three abovementioned datasets are listed in Table 1. In total, our merged dataset comprises 101 countries (see Table 2) and spans from March 2020 to March 2021, encompassing the first year since the pandemic has been declared.

**Table 2:** Countries analysed.

Continent	Countries
Africa	Kenya, Tanzania, South Africa, Mozambique, Morocco, Malawi, Libya, Ghana, Ethiopia, Egypt, Cape Verde, Burkina Faso, Zambia, Algeria
Asia	Malaysia, Iraq, Iran, Indonesia, India, Pakistan, Philippines, Nepal, Azerbaijan, Japan, Jordan, Saudi Arabia, Georgia, Kazakhstan, Singapore, Vietnam, Kuwait, South Korea, Bhutan, Sri Lanka, Lebanon, Bangladesh, Thailand, China, Turkey, United Arab Emirates, Israel
Europe	Austria, Moldova, Malta, Netherlands, Bulgaria, Poland, Romania, Russia, Serbia, Slovakia, Slovenia, Spain, Sweden, Switzerland, Ukraine, United Kingdom, Norway, Portugal, Luxembourg, Belgium, Bosnia and Herzegovina, Croatia, Denmark, Estonia, Finland, France, Germany, Belarus, Greece, Hungary, Albania, Lithuania, Iceland, Ireland, Italy, Latvia
North America	Mexico, Dominican Republic, United States, El Salvador, Trinidad and Tobago, Canada, Panama, Guatemala, Honduras, Jamaica, Costa Rica
South America	Uruguay, Venezuela, Bolivia, Paraguay, Colombia, Peru, Ecuador, Argentina, Brazil, Chile, Suriname
Oceania	New Zealand, Australia

We first analyzed the influence of demographic variables, also including the cultural dimensions (identified in the categories “demographic” and “cultural context” in Table 1) in the six COVID-19 metrics highlighted in Table 1. Pearson's correlation coefficient is usually adopted for variables with normal distribution (Schober and Schwarte, 2018). Thus, we adopted a similar procedure to the one followed by Teh et al. (2021) and computed the Pearson correlation to assess the correlations between each pair of variables. Given the large number of variables, to facilitate the visualization, we plotted a heatmap with colors emphasizing the strength of each correlation. Additionally, considering existing literature acknowledges the impact of NPIs in mitigating the COVID-19 effects, we also plotted a heatmap that correlates each NPI variable and each of six COVID-19 metrics, to understand if the demographic variables and, especially, the cultural dimensions play an equally important role in explaining COVID-19 effect when compared to NPIs.

## 3 Results and discussion

**Figure 1:**
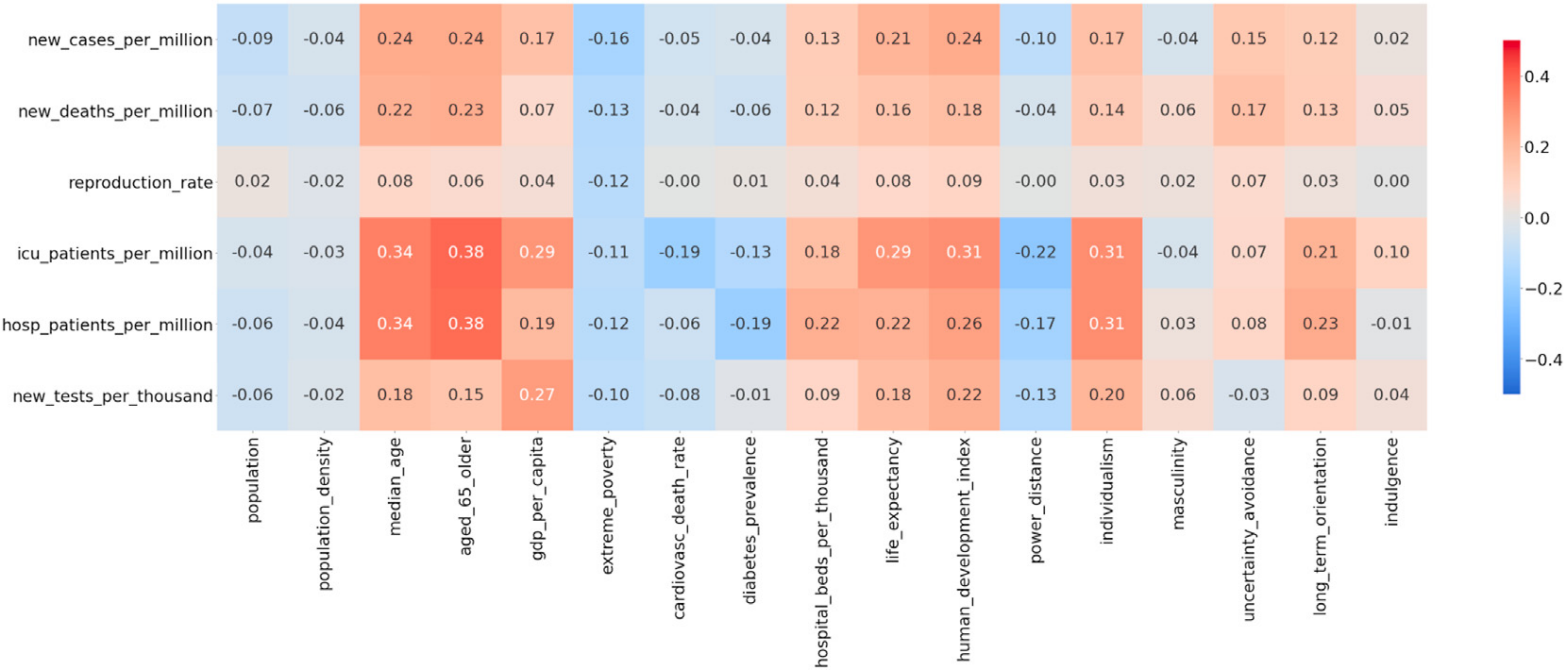
Heatmap for demographic and cultural dimensions.

Figure 1 shows the correlation heatmap for demographic and cultural dimensions. The rows represent the six COVID-19 metrics (dependent variables), while the columns represent the demographic and cultural variables (independent variables). Thus, each cell contains the computed Pearson correlation value between the column's independent variable and the row's dependent variable. A negative value implies that the correlation is in the opposite direction, i.e., as the independent variable increases, the dependent variable decreases. We consider the benchmarks proposed by Cohen (2013) who highlights that a small correlation comprehends an absolute value between 0.1 and 0.3, whereas a moderate correlation ranges from 0.3 to 0.5, while a large correlation includes values between 0.5 and 1. However, Hemphill (2003) argues that the thresholds proposed by Cohen (his first edition is of 1977) are higher than needed for practical studies where somewhat small correlations from most statistical standards provide important takeaways (he cites the example of Rosenthal, 1991, who found a correlation of 0.3 between taking aspirin and preventing heart attacks).

For an easier visualization, we coloured the cells in blue tones (negative correlations) or read tones (positive correlations), with a stronger tone implying a stronger correlation. Some cells immediately emerge from Figure 1. Specifically, both median age and people with 65 years age or older have the largest correlation with both intensive care unit (ICU) and hospitalized patients. This is aligned with existing literature, that acknowledges elderly as the most severely affected by the COVID-19 disease, with devastating consequences in nursing homes (Lopez and Neely, 2021). As for the remaining social and demographic variables, we can observe that the higher the GDP, the higher the number of ICU patients. This is most likely the effect of richer countries offering a larger number of ICU beds in hospitals. Likewise, countries with higher GDPs can test more. Also, both a higher life expectancy and human development index positively influences most COVID-19 metrics, e.g., the higher the GDP, the higher the number of available beds.

**Figure 2:**
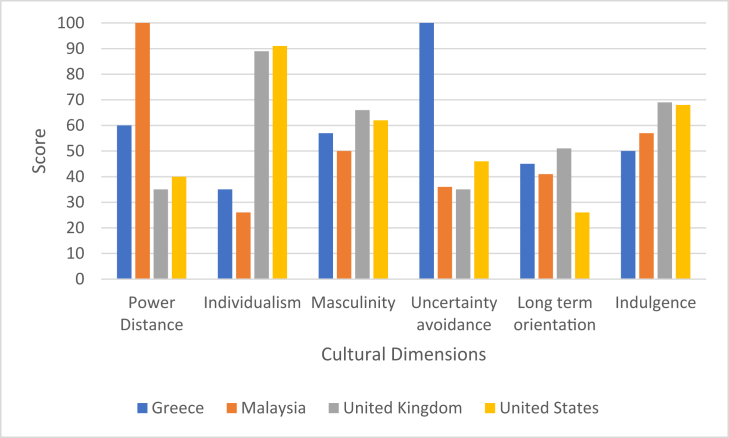
Examples of cultural dimensions.

Figure 1 denotes that while some cultural variables do not play a significant role in COVID-19 metrics, others do show moderate correlations. Examples of the former are masculinity and indulgence. According to Hofstede et al. (2010), masculinity indicates a society that is driven by success, rather than by values related to attaining a sustainable quality of life. The obtained results show that the goal of fighting COVID-19 and mitigating its effects are independent of the society's competitiveness in terms of the time and effort spent in labor versus home. However, several of the cultural variables show weak to moderate correlations with some of the COVID-19 metrics. Examples (shown on Figure 2) are countries with lower power distance (e.g., United States, United Kingdom) show higher numbers of patients (both hospitalized and in ICU) when compared to countries with higher power distance (e.g., Malaysia).

Citizens in countries showing a large power distance seem to accept more easily a hierarchical order in which everybody has its place and which requires no further justification, while in countries exhibiting low power distance, societal forces are under strain to try to balance the distribution of power and plead justification for inequalities of power. While one could argue that some of the Western countries (with low power distance) that have been more severely affected have a higher capacity in number of beds to receive patients, which might signal that this result is due to the maturity of national healthcare systems, we highlight that Eastern countries such as China, with a developed healthcare system, also have high power distance. Thus, such result can suggest that, in countries with high power distance, there is more respect for national authorities (including health professionals) and abidance to the limitation rules, which can lead to a higher degree of commitment to the restriction rules, ultimately resulting in a lower degree of the pandemic severity.

A similar rationale can be built for the individualism dimension, which denotes societies where each individual is supposed to lookup for himself/herself and direct family only, versus collectivist societies where people belong to larger groups that are supposed to take care of the whole group, to which their members are loyal (Hofstede et al., 2010). Interestingly, individualism is among the variables with the highest correlation with COVID-19 metrics, namely the number of ICU and hospitalized occupied beds variables, as shown on Figure 1.

Our results show that individualist societies tend to have higher numbers of hospitalized patients, including in ICU. Also, there is a weak correlation with the number of new cases and new deaths.

**Figure 3:**
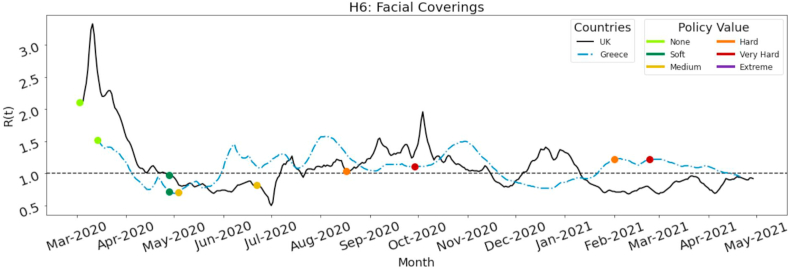
Facial coverings restriction policy and Rt evolution for the UK and Greece.

**Figure 4:**
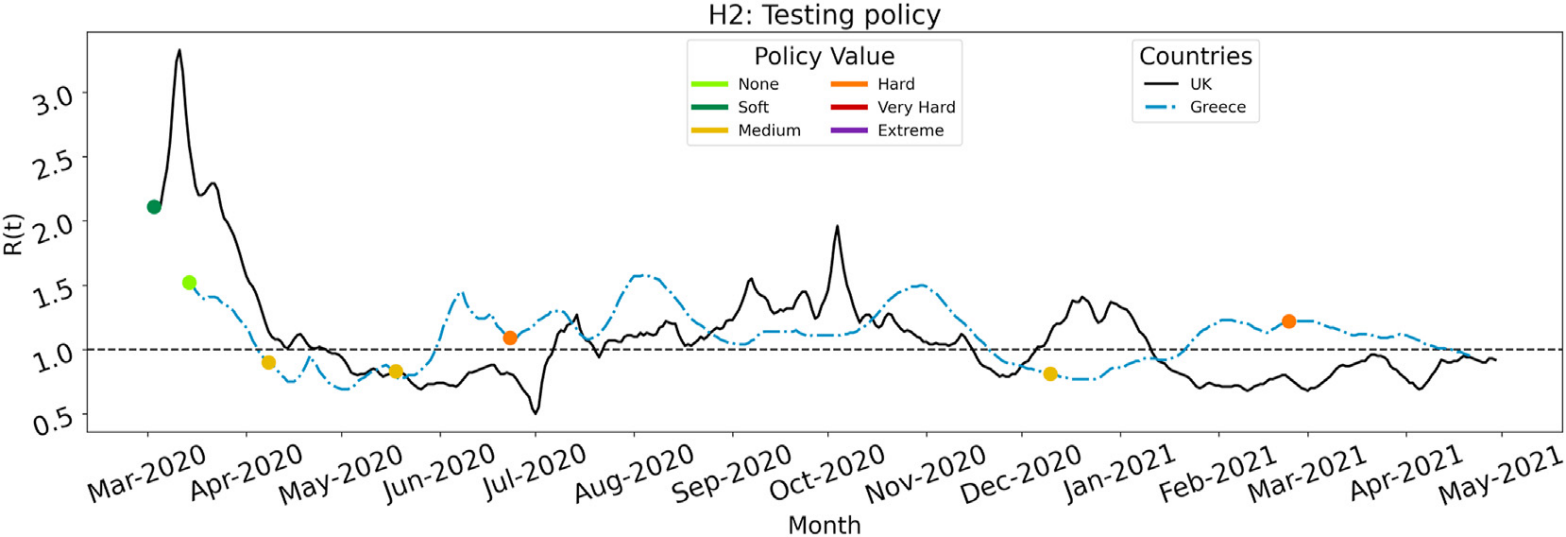
Testing policy restriction policy and Rt evolution for the UK and Greece.

**Figure 5:**
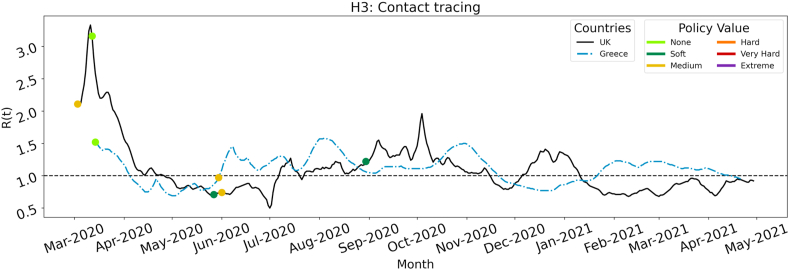
Contact tracing restriction policy and Rt evolution for the UK and Greece.

This finding suggests that citizens in societies that are not devoted to thinking in benefit of the larger group are only concerned in protecting themselves and not showing particular concern outside their direct family. On the opposite, in countries with lower individualism such as Greece (Figure 2), governments continue to adopt more cautious policies, still enforcing several restrictions such as mask wearing, despite on-going vaccination campaigns (U.S. Embassy & Consulate in Greece, 2021). By comparing Greece and UK (Figure 3), we can observe that the Rt is smoother and more stable (i.e., with lower variations) in Greece when compared to the UK. Even so, Figure 3 shows that Greece has imposed most of the time a very hard restriction policy regarding facial coverings. On the opposite, the UK has imposed hard (instead of very hard) facial coverings policy. In addition, other health NPIs were kept with a high degree of restriction over time such as contact tracing and policy testing. In Figure 4 we can see that in Greece the testing policy was implemented with hard degree when the Rt was above 1 while in the UK this NPI was always kept at a medium degree from May 2020 until May 2021, even when the Rt was way above 1. While in Greece the contact tracing policy was implemented at a medium degree from late May 2020 (Figure 5), in the UK this NPI was implemented at a medium degree from June to September 2020 and then relieved to a soft degree and kept at this degree even when the Rt had a peak at the beginning of October 2020. One could state the conjunction of Greece's cultural dimensions of being a society that stands out as highly collectivist and that avoids uncertainty contrasts with a more individualistic and much less uncertainty avoidant society in the UK.

**Figure 6:**
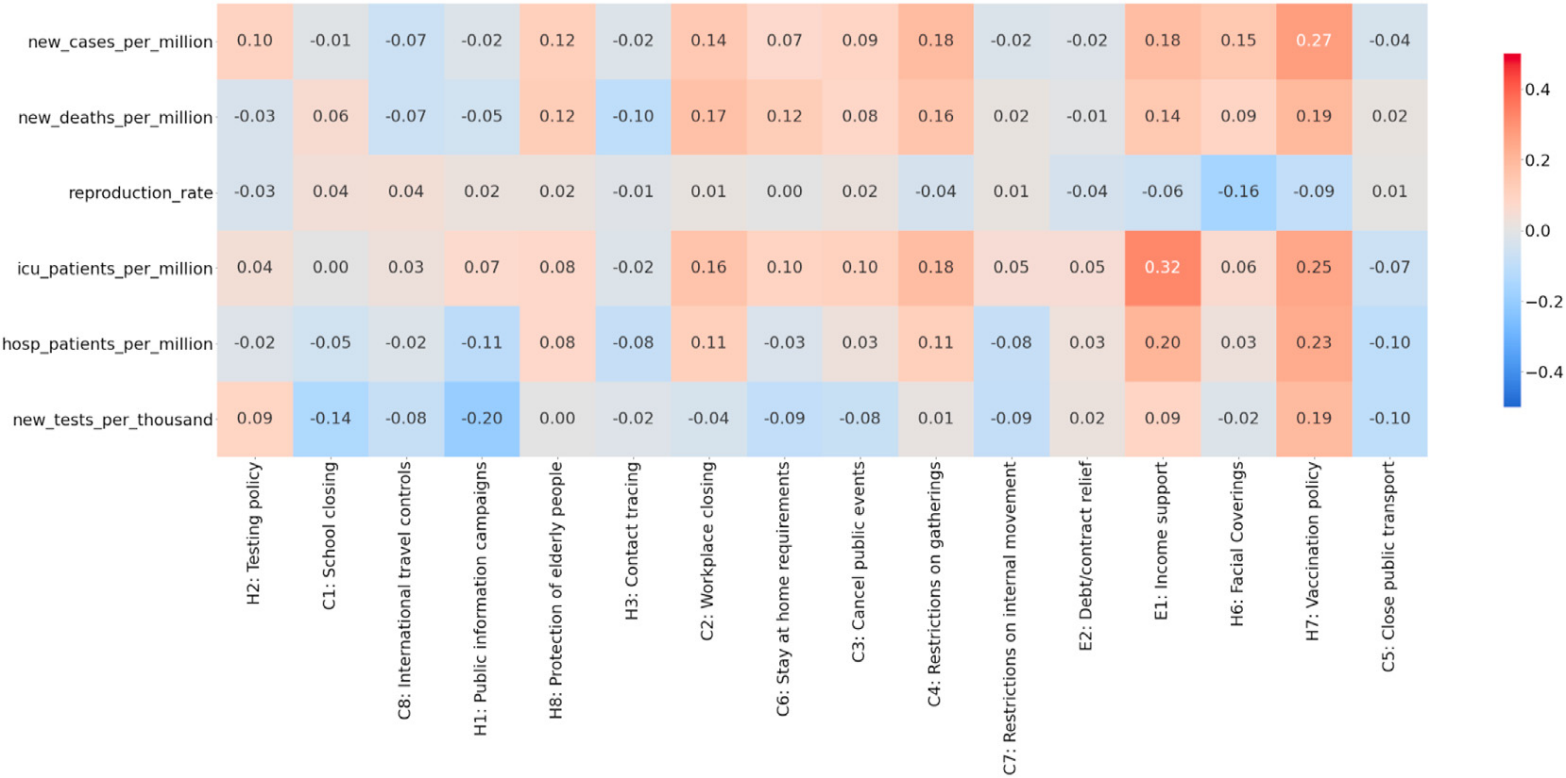
Heatmap for NPIs.

By comparison, we also plotted the heatmap for the NPI variables in Figure 6. We emphasize that existing literature acknowledges the influence of combined NPIs on COVID-19 mitigating impact (e.g., Haug et al., 2020; Bendavid et al., 2021). Nevertheless, several of the cultural dimensions (i.e., power distance, individualism, uncertainty avoidance, and long-term orientation) show equal or higher values of correlation in comparison to most of NPIs identified in Figure 6. Such finding unveils that both scholars and practitioners aiming to model the COVID-19 evolution to predict the future impact of infectious diseases pandemics at a worldwide scale should account not only to social/demographic and NPI variables, but also include the cultural dimensions.

## 4 Conclusions

As the COVID-19 continues to spread havoc throughout the globe, both scholars and practitioners struggle to improve the accuracy of data-driven models that can predict this pandemic evolution or future infectious diseases pandemics. So far, most researchers have focused on social and demographic variables, and in the implemented NPIs to mitigate the disease dissemination. In this study, we argue that the cultural context also plays a role. Specifically, we considered the Hofstede's six dimensions when analyzing data from many countries worldwide, with four of these cultural dimensions showing correlations with several of the COVID-19 impact metrics. Our findings suggest a promising avenue of future research by proposing including variables that reflect the communities' cultural context in COVID-19 predictive models. Nevertheless, the widely adopted Hofstede's dimensions can only be used by assessing several countries in the same model. This is an important limitation that can be overcome by proposing higher granularity cultural variables. Furthermore, our analysis is limited due to the difficulty of distinguishing between choosing not to comply and not being able to comply with NPIs e.g., it is not possible for everyone to work from home. Also, the level of each NPI is not the same across the countries, and while there was an effort of normalizing NPI levels by the authors of the OxCGRT dataset, the granularity of the levels cannot fully reflect the differences among countries in the same NPIs. For example, C7, restrictions on internal movement, has only 3 levels: 0 - no measures; 1 - recommend not to travel between regions/cities; 2 - internal movement restrictions in place. Thus, if a country places a restriction to only each resident's neighborhood, that is not reflected on the C7 NPI computed. Additionally, the reliability of the metrics reported depend on national entities, which may have or not integrated systems to account for COVID-19 numbers.

As our study considers daily temporal snapshots of the collected variables, while we are able to assess the correlations of cultural dimensions and NPIs to COVID-19 metrics, we cannot directly infer if we could predict the pandemic's evolution overtime. To address this limitation, we intend to conduct future research to develop a data-driven model based on time-series analysis that considers past pandemic situation to predict potential future ones, besides the NPIs implementation and cultural and demographic dimensions.

## Declarations

### Author contribution statement

Margarida Duarte: Conceived and designed the experiments; Performed the experiments; Analyzed and interpreted the data; Wrote the paper.

Sérgio Moro and Catarina Ferreira da Silva: Analyzed and interpreted the data; Wrote the paper.

### Funding statement

This work was supported by the Fundação para a Ciência e Tecnologia (FCT) within the following Projects: UIDB/04466/2020 and UIDP/04466/2020.

### Data availability statement

Data will be made available on request.

### Declaration of interests statement

The authors declare no conflict of interest.

### Additional information

No additional information is available for this paper.

## Footnotes

1 https://www.hofstede-insights.com/.

2 https://ourworldindata.org/coronavirus-source-data.

3 https://github.com/OxCGRT/covid-policy-tracker.
